# Radiation-induced cutaneous vasculopathy of the breast: a rare case report

**DOI:** 10.1186/s12957-024-03346-0

**Published:** 2024-02-21

**Authors:** Hilde Van Parijs, Yves Sinove, Marilyn Carprieaux, Mark De Ridder

**Affiliations:** 1 Department of Radiotherapy, UZ Brussel, Vrije Universiteit Brussel, Laarbeeklaan 101, Brussels, 1090 Belgium; 2 Department of Plastic Surgery, A.S.Z, Merestraat 80, Aalst, 9300 Belgium; 3 Department of Pathology, A.S.Z, Merestraat 80, Aalst, 9300 Belgium

**Keywords:** Breast cancer, Radiation therapy, Vasculopathy, Purpura, Ecchymosis, Late toxicity

## Abstract

**Background:**

Radiation therapy is often indicated as part of the treatment for breast cancer and is therefore used frequently worldwide. Vasculopathy is a general term used to describe any condition that affects blood vessels. We present a case report of a patient who presented with vasculopathy as a rare late side effect of radiation therapy to the breast.

**Case presentation:**

This 66-year-old woman was initially treated with breast-conserving surgery for early-stage receptor-positive left breast carcinoma. She received postoperative radiation therapy and hormonal treatment with tamoxifen. She developed sudden spontaneous painless ecchymosis spread over the whole irradiated area 1.5 years after finishing her radiation therapy. Tumor relapse was excluded. There was no associated vasculitis. The cause was presumed to be multifactorial. She had a history of smoking and was known to have hyperlipidemia. She had undergone several surgical treatments at the left breast one year after her initial breast-conserving treatment and was taking tamoxifen. Anti-inflammatory medicine and treatments increasing local blood flow were prescribed. The ecchymosis resolved completely within one month.

**Conclusions:**

Vasculopathy can occur as a rare late side effect of radiation therapy. It can be reversible. Prevention begins with carefully treating precipitating factors.

## Background

Radiation therapy (RT) of the breast is a standard treatment after breast-conserving surgery and is often indicated after mastectomy or in patients with lymph node involvement [1–3]. The most common acute side effects occur on the skin. These lesions appear within days to weeks and heal easily and completely. Chronic effects can take months to years to manifest. Late skin toxicity, such as hyperpigmentation, telangiectasia, and atrophy, is often observed and is mostly harmless but permanent. We report a case of sudden vasculopathy as a rare, late side effect of RT to the breast. To our knowledge, no similar case has been reported before.

## Case presentation

**Fig. 1:**
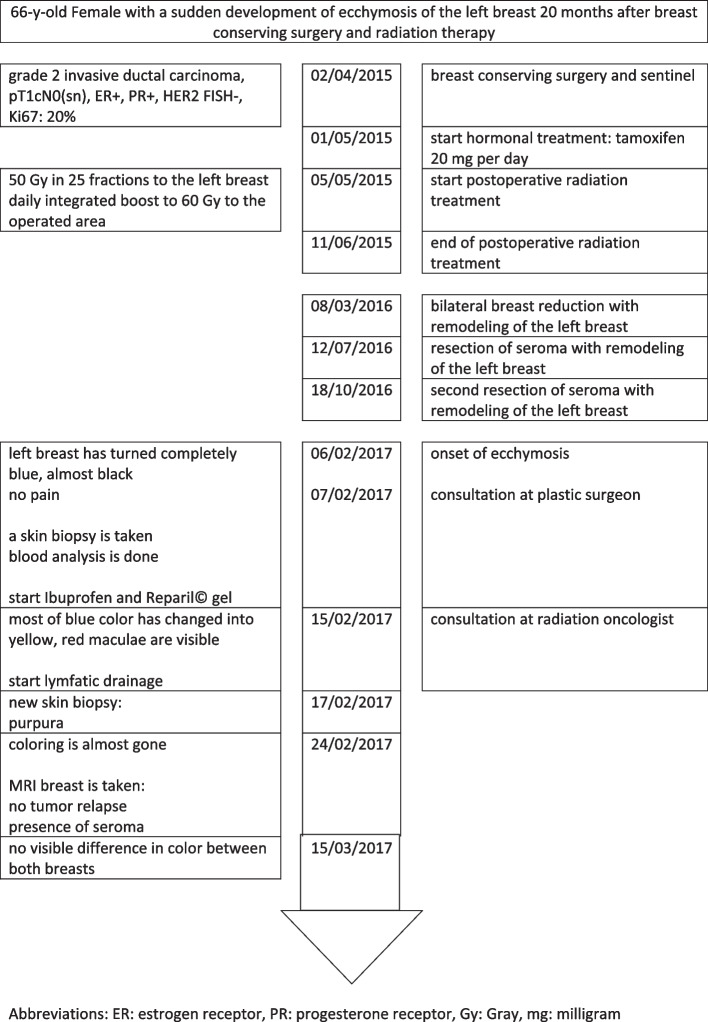
Patient timeline

**Fig. 2:**
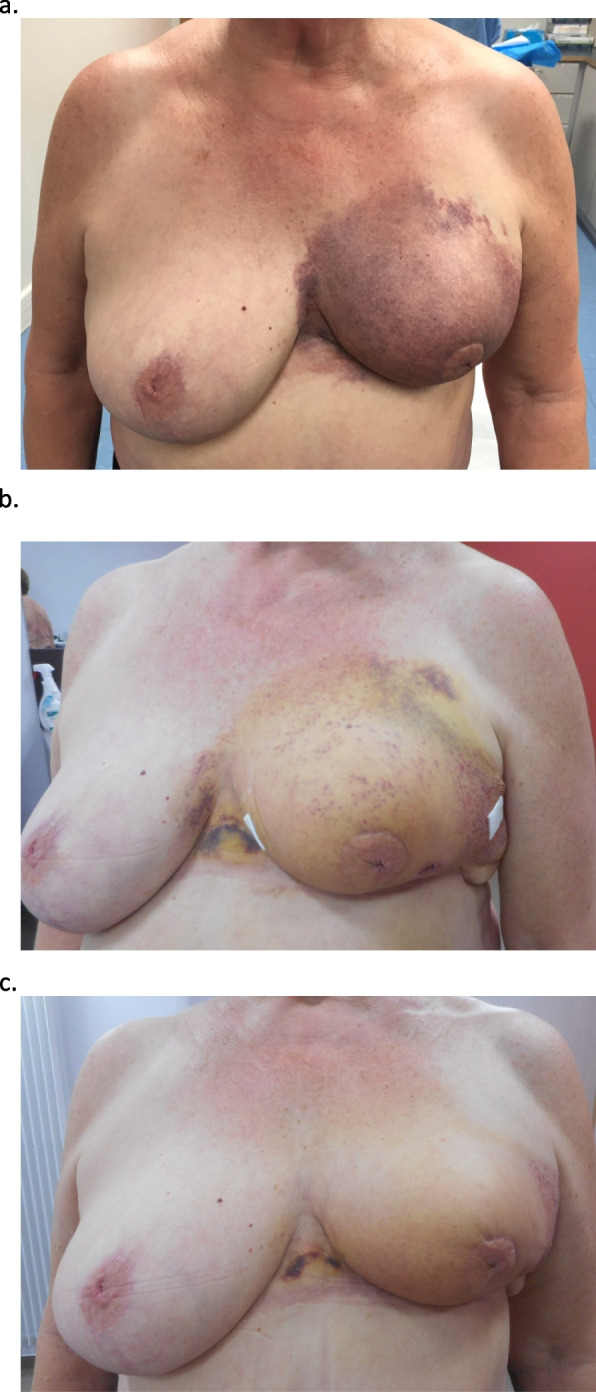
Pictures. **a** Onset of ecchymosis, **b** After 1 week: the blue color had largely changed into yellow, purpura are present, known teleangiectasia are visible, bandages after biopsy, **c** After 2 weeks: most of the ecchymosis has resolved

A 66-year-old woman presented to her plastic surgeon a few days after she awoke one morning and noticed that her left breast had turned completely blue and almost black (Figs. 1 and 2a). There was no associated pain. There was no history of an accident or impact on the breast.

Two years earlier, she underwent breast conserving surgery with sentinel lymph node biopsy (sn) for invasive ductal cancer located in the upper external quadrant of the left breast, stage pT1cN0 (sn). Estrogen and progesterone receptor status was positive. There was no distant metastasis. Surgery was performed uncomplicatedly, and her wounds healed well. Hormonal treatment with 20 milligrams (mg) of tamoxifen per day was started. She received postoperative RT to 50 Gy (Gy) in 25 fractions to the whole left breast with a simultaneous integrated boost of 0.4 Gy to the operated area to a total dose of 60 Gy, as was the standard dose prescription at that time. No unexpected acute side effects occurred. A localized moist desquamation of the skin at the boost area was present shortly after terminating RT and resolved quickly by applying hydrating crème. In the long term, she developed gross telangiectasia in the boost area.

According to her relevant previous history, she was diagnosed with sarcoidosis in 1978, during which she received corticosteroids for more than two years. In 1984, she needed corticosteroids again for skin sarcoidosis, and in 1989, she needed corticosteroids for liver sarcoidosis. She developed erythema nodosum in 1990. Corticosteroids were stopped in 1991. She had smoked cigarettes until 1990. She was treated for hypertension with 100 mg of atenolol per day and for hypertriglyceridemia with 10 mg of atorvastatin per day.

At the time of her breast cancer treatment, the patient had a large breasts, and the bra-size was the G-cup. One year after her breast cancer diagnosis, she underwent bilateral breast reduction. Four months later, she presented with a nodule in the left breast, which appeared to be a seroma. This was resected, and remodeling of the left breast was performed. Three months later, a new seroma had developed in the left breast that needed to be resected. Again, there was remodeling of the breast. At both times, the drain was left for several days.

The described episode occurred 4 months after this last intervention. The distinct bluish color was distributed throughout the whole breast and clearly delineated. The surgeon suspected a possible relationship with the delivered RT. Ibuprofen and a gel containing aescine and salicylate were prescribed. Blood analysis was also performed. Hematology, including leucocyte formula, revealed elevated reticulocytes (27/1000, reference 5–20/1000) and no other abnormalities. Coagulation factor levels, glycemia, iron status, renal function, inflammatory factor levels, pancreatic enzyme levels, and thyroid function were normal. Her liver enzymes revealed a slightly elevated γGT (42 U/L, reference 5–36 U/L), and the other liver enzymes were within the normal range. A high level of triglycerides (218 mg/dL, reference < 150 mg/dL) was present, with normal cholesterol. A full-thickness skin biopsy showed ectatic venules at the derm with intraluminal fibrin thrombi, as is observed in vasculopathy. There was a slight perivascular lymphocytic infiltrate. The epidermis and hypoderm were normal.

**Fig. 3:**
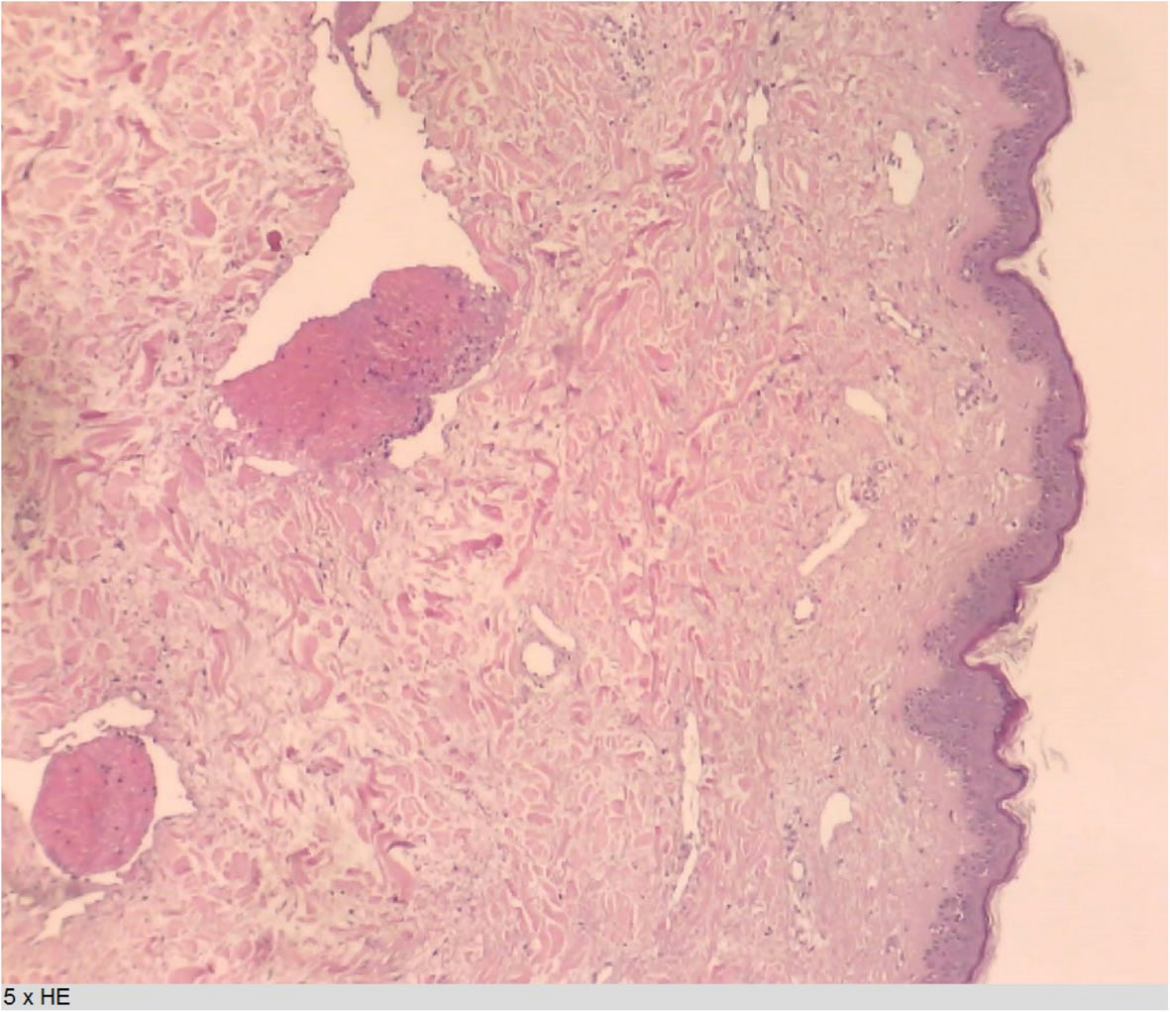
Light microscopy of derm with ectatic venules and intraluminal thrombi

One week later, the blue color had largely changed to yellow (Fig. 2b). Red macula were visible. Gross telangiectasia was present around the boost area and in the inframammary fold. Their presence was known prior to this event. New full-thickness skin biopsies were performed, revealing a normal epidermis but dilated vessels and extravasation of red blood cells in the derm (Fig. 3). This led to the diagnosis of purpura. There were no signs of vasculitis. No tumor cells were observed. Manual lymphatic drainage of the breast was added to the treatment.

During the second week, a magnetic resonance imaging scan of the breasts was performed, excluding breast cancer relapse but revealing the remnant of a known seroma. At this time, the hematoma had almost completely vanished (Fig. 2c). The patient mentioned that lymphatic drainage offered relief.

One month after onset, the ecchymosis had completely resolved. The skin color no longer differed from that of the contralateral breast.

## Discussion

### Understanding radiation-induced vascular disease

Vasculopathy is a general term used to describe any condition that affects blood vessels. Purpura and ecchymoses are caused by extravasation of blood from the vasculature into the skin. The differential diagnosis can be divided into platelet disorders, vascular factor deficiencies and coagulation factor deficiencies. Disorders can be congenital. They can be associated with vasculitis or can be caused by trauma, medications, infections, or malignancy.

Radiation-induced vascular disease after RT to the heart, neck or brain is well known because of its serious, possibly fatal consequences. The clinical manifestations vary from coronary insults and myocardial infarction to heart failure and stroke. Pathology of these manifestations progresses slowly, and the duration from RT to clinical manifestations can exceed 10 years [4–6]. The cause of radiotherapy-induced coronary heart disease is suggested to be the induction or acceleration of atherosclerosis in conduit arteries located in the irradiated field [7]. The incidence is greater in patients with ‘classical’ risk factors, such as smoking, hypertension and obesity [8]. After RT of the neck, Silverberg et al. described in 1978 a pattern of atherosclerotic changes on angiography of the carotid arteries, even in areas unusual for the natural occurrence of arterial disease. Patients showing radiation-induced atherosclerosis were significantly younger and had significantly fewer generalized lesions than patients showing carotid vascular disease without associated RT [9]. In the brain, vasculopathic changes are thought to be a central diagnostic feature of late radiation injury. Wang et al. found 77 cases of delayed radiation-induced cerebrovasculopathy after pediatric intracranial irradiation [10]. There was a statistically significant correlation between increasing doses of radiation and earlier presentation.

The mechanism of radiation injury is similar in all blood vessels and has been linked mainly to endothelial dysfunction [11, 12]. Initial endothelial loss is followed by and partially overlapped by thrombi formation and hemorrhage. Long-term morphological changes include endothelial proliferation, basement membrane thickening, adventitial fibrosis, and vessel dilatation [13]. Among all blood vessels, capillaries are the most radiosensitive because they have only a single layer of endothelium. Well-differentiated endothelial cells, as found in dermal capillaries, undergo cellular senescence similar to aging and premature atherosclerosis. Senescence induced by DNA damage from irradiation could lead to slower growth and perhaps increased vascular permeability [14].

Microvascular dysfunction has been demonstrated [15]. This could be explained by the fact that irradiated tissues suffer from chronic oxidative stress accompanied by increased production of reactive oxygen species [16]. Overproduction of reactive oxygen species is also regarded as an integral part of atherosclerosis [17]. In vitro studies have suggested that radiation induces endothelial activation characterized by activation of the transcription factor nuclear factor kappa beta (NF-κB), resulting in alterations in vascular adhesion molecule expression and chemokine and cytokine production [18–21]. The activated endothelium is prothrombotic as a result of leucocyte-endothelial cell or platelet-endothelial cell adherence, leucocyte infiltration into tissue and thrombus formation [22–24]. By comparing irradiated arteries with nonirradiated arteries from the same patient, it has been possible to confirm NF-κB activation by RT in humans [25]. Activation of NF-κB is regarded as one of the most important and early events in endothelial activation [26]. Leukocyte adhesion to endothelial cells and thrombi can block the vascular lumen, as can the growth of endothelial cell colonies during vascular regeneration [27–29]. These alterations cause chronic injury to endothelial cells, which can lead to visible changes over months to years. Fajardo described the morphologic patterns of the effect of radiation on mammalian tissues [30]. Radiation does not produce pathognomonic morphologic features. However, a consistent feature is the lack or paucity of a cellular inflammatory response. The most radiosensitive blood capillaries and sinusoids can exhibit irregular cytoplasm with the formation of pseudopodia, swelling of ‘blebs’ in the cytoplasm, detachment of endothelial cells from the basal lamina, cell pyknosis, rupture of the plasma membrane, thrombosis, and rupture of the capillary wall.

### Causes and precipitating factors

The risk and severity of late reactions depend on several factors. Radiation-related treatment factors included the total dose, the dose per fraction, and the schedule of treatment. Late effects are generally more sensitive to changes in fraction size and less sensitive to changes in overall treatment time [31, 32]. When a relatively large dose of radiation is administered, blood vessels tend to develop edema, thrombosis, and hemorrhage. In contrast, when a lower dose of radiation is given, vascular injury is not initially evident but rather manifests as delayed telangiectasia formation and hemorrhagic infarcts until 1–2 years after the completion of radiation exposure [30, 33].

Patient-related factors include age at the time of RT, trauma, or surgery at an irradiated site and comorbidities, particularly those involving impaired vascularity, such as diabetes and hypertension [34, 35]. Patients with scleroderma and systemic lupus erythematosus are at increased risk of severe toxicity [36].

### About this patient

Several factors could have triggered this incident. She had developed widespread telangiectasia, a sign of radiation-induced injury to the vessels of the irradiated breast. Blood analysis revealed an elevated level of serum lipids even though she was taking atorvastatin. Tamoxifen, which is known to be thrombotic, was used as part of her treatment. She had a history of smoking, although she had stopped smoking more than two decades ago. There was a history of skin sarcoidosis. She had undergone multiple surgical interventions in the treated breast.

The possible presence of a platelet disorder, a vascular factor, or a coagulation factor deficiency was ruled out as a precipitating comorbidity by history, clinical examination, and blood analysis. She did not have any congenital disorders that could be associated with vasculitis. No recent trauma or infection had occurred. Tumor relapse was not observed via magnetic resonance imaging of the breast.

However, which factor caused the sudden unset of this vasculopathic reaction is unclear. Most likely, this incident was multifactorial.

### Treatment

Treatment of radiation-induced vasculopathy depends on the severity of the condition. For nonlife-threatening patients, treatment usually focuses on using medication to relax blood vessels and allow better blood flow. Medication can be prescribed to help prevent blood clots from forming. Radiation-induced atherosclerosis and stenosis of large vessels are treated as nonradiation-induced lesions without increased mortality [9].

## Conclusions

The pathological processes of radiation injury are complex and begin immediately after radiation exposure, but the clinical and histological features may not become apparent for weeks, months, or even years after treatment. When patients present with radiation-induced vasculopathy, a thorough review of possible precipitating factors and exclusion of tumor relapse should be performed. Sufficient treatment of the known risk factors for atherosclerosis may be important for limiting late toxicity. Cutaneous vasculopathy can be reversible.

## Data Availability

All data uses for this case report can be found in the electronic files of the patient at A.S.Z. Aalst and UZ Brussel, Belgium.

## Abbreviations

RT: Radiation therapy
sn: sentinel node biopsy
mg: milligrams
Gy: Gray
U/L: units per liter
dL: deciliter
NF-κB: nuclear factor kappa B
